# Microsphere Coupled Off-Core Fiber Sensor for Ultrasound Sensing

**DOI:** 10.3390/s22145328

**Published:** 2022-07-16

**Authors:** Gerard Tatel, Xiaoyi Bao

**Affiliations:** Physics Department, University of Ottawa, Ottawa, ON K1N 6N5, Canada; gtate010@uottawa.ca

**Keywords:** Fabry–Perot interferometer, microsphere, ultrasound, off-core, finesse

## Abstract

A compact fiber ultrasound-sensing device comprising a commercially available Barium Titanate (BaTiO_3_) glass microsphere coupled to an open cavity off-core Fabry–Perot interferometer (FPI) fiber sensor is proposed and demonstrated. The open cavity is fabricated through splicing two segments of a single mode fiber (SMF-28) at lateral offsets. The lateral offset is matched to the radius of the microsphere to maximize their coupling and allow for an increased sensing response. Furthermore, the microsphere can be moved along the open-air cavity to allow for tuning of the reflection spectrum. The multiple passes of the FPI enabled by the high refractive index microsphere results in a 40 dB enhancement of finesse and achieves broadband ultrasound sensing from 0.1–45.6 MHz driven via a piezoelectric transducer (PZT) centered at 3.7 MHz. The goal is to achieve frequency detection in the MHz range using a repeatable, cost effective, and easy to fabricate FPI sensor design.

## 1. Introduction

Fiber optic interferometric sensors have gained interest in recent times due to their distinctive characteristics, such as compact size, repeatability, and multi-parameter sensing capabilities. Due to their diverse sensing applications and inexpensive designs, these sensors have applications in strain monitoring [1,2,3], refractive index measurements [4,5,6], and temperature [7,8,9]. More specifically, Fabry–Perot interferometers (FPI) have been investigated thoroughly due to desirable characteristics, such as high finesse-associated narrow spectrum peaks and high sensitivity, compact size, and immunity to electromagnetic interference. FPI are typically formed by cascading reflective surfaces or reflectors along the light’s propagation path and their interference. Fiber optic FPI sensing devices can be divided into two subcategories: intrinsic FPIs, where light interactions are inside of the fiber, which can be formed through techniques, such as thin film deposition [10]; Bragg gratings [11]; and micro machining [12], and extrinsic FPIs, where light interacts with external cavities, such as air or other polymers [13]. These open-air cavity FPIs are typically fabricated through expensive and complex methods, such as fs laser micro-machining [14]. The sensing capabilities of these type of fiber optic sensors have been exploited and enhanced through new and old fiber optic technology innovations, such as fiber grating [15,16,17], surface plasmon resonance [18], and specialty fibers [19].

**Figure 1:**
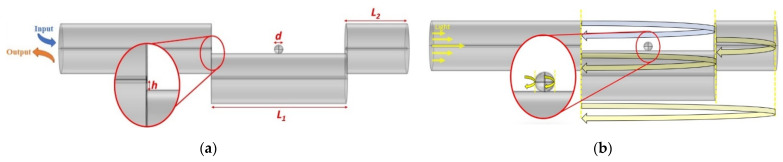
(**a**) 3D representation of the proposed sensor. (**b**) Multiple FPI paths depicted by curved arrows; the blue arrow depicts the path through air exclusively.

To mitigate the cost for these open-air cavity sensing devices, splicing two sections of single mode fiber segments at a lateral offset to form a simple and compact sensor has been explored [20,21,22]. Splicing several offset segments beyond two sections has also been explored [23]. At each silica–air interface there will be a reflection, essentially turning the device into multiple cascading FPIs. The large offset of this FPI allows light to spread from the main incoming fiber to both the offset cladding and the surrounding open-air cavity, this will yield two definitive interferometer arms with a large refractive index difference. These types of open cavity FPIs have been demonstrated to have a diverse range of sensing capabilities, such as temperature [24], refractive index [25], stress and strain [26,27], and ultrasound detection. For ultrasound detection, Fan et al. detail that the importance of high reflectivity, relatively large contrast, and linewidth would benefit the high frequency ultrasound sensor response [28]. Because the displacement of ultrasound sensing at MHz can be as small as sub-um [29], which is impossible to be detected with interferometers due to the diffraction limit of the laser wavelength, to increase the detection sensitivity for intensity detection, the sensor must operate at a quadrature point where relative intensity change versus wavelength shifts due to the displacement of the ultrasound signal, defined as intensity slope. To increase the sensitivity of the ultrasound sensing at the highest frequency, the intensity slope should be at its maximum. To improve this slope for FPI, the finesse is proportional to intensity slope change. For a fixed reflectivity and cavity length, the spectrum of the FPI exhibits narrow linewidth with a high finesse ℱ. Light propagates in more FP cavities representing a higher finesse ${ℱ}^{N}$ contributed to the product of the finesse of *N* individual FPIs with the same resonant frequency, these individual FPIs are represented by curved arrows in Figure 1b. To reach this target, we added a microsphere, as shown in Figure 1a, with a high refractive index between two sections of the off-core fiber (open cavity); through the front and back surfaces of the microsphere, the first FPI reaches the double pass, and the same principle is applied to the second FPI, the front and back surface of the microsphere introduced the 2nd double pass FPI, which leads to a total of ${ℱ}^{6}$, which can be seen in Figure 1b, circled in red. In addition to the FPI finesse enhancement, the microsphere itself is a resonator device, which enables light to be stored and confined at a resonant frequency. This confined light will circulate within the device through total internal reflection.

In this paper, an extrinsic FPI fiber sensor is proposed and fabricated by the lateral offset splicing of two single mode fiber segments (Corning SMF-28) acting as the base structure, which is then modified and enhanced by utilizing the benefits of increased finesse by adding a Barium Titanate (BaTiO_3_) glass microsphere (refractive index of 1.94, commercially available from Cospheric LLC) coupled in the open-air cavity. The lateral offset and microsphere diameter are carefully matched to achieve the maximum contrast; this will allow for an increase in sensitivity. Light is reflected at each air–silica boundary. From the reflection spectrum, quadrature points are selected due to the wavelength shift-induced maximum intensity change and are used for ultrasound detection. The fabricated sensor is tested with an ultrasound source generated from a piezoelectric transducer (PZT) centered at 3.7 MHz attached to a thin steel plate with the excitation of its high-order harmonics to increase the ultrasound frequency range.

## 2. Materials and Methods

**Figure 2:**
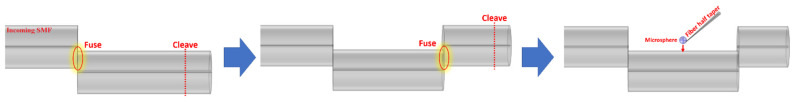
Fabrication process of the proposed sensor.

A visual representation of the proposed sensing device is illustrated in Figure 1a. To begin, after prepping two SMF segments for splicing and loading it into the fiber splicer (Ericsson Cables, Sundbyberg, Sweden, FSU 995 FA), manual mode is selected. Using the two views in the splicer (side and top view), the top view is aligned (via lining up the SMF core) while the other is set to an offset distance *h* (the distance from the incoming SMF center point of the core to the 1st segment SMF’s edge), circled in red in Figure 1a. The offset is imperative as it will affect the beam path through the air and microsphere interfaces, which is vital for characteristics, such as contrast and reflectivity. Once the leading fiber and 1st segment is fused, it is carefully cleaved under a confocal microscope to the desired length *L*_1_. This length will be the air cavity enclosed by two SMF segments (incoming SMF and end segment SMF). Similarly, the end segment is spliced and fused to the previous SMF segment, making sure all the cores align in the top view. Once the end segment is matched to the same offset it is spliced and fused; it is brought under the confocal microscope again for the final cleave at the desired length *L*_2_. Under the microscope and shielded from the environmental effects, a single BaTiO_3_ microsphere is selected and picked using fabricated fiber half tapers with a waist diameter of ~5 µm. Combining with translational stages at different positions, the microsphere is deposited onto the open-air cavity, and its position can be moved using the same fiber tapers. This process is illustrated in Figure 2.

In the most basic case (just the off-core), we can analyze the sensor as cascading FPIs and derived with 2-beam approximation with three mirrors. The total reflected electrical fields is approximated as *E_r_* given by [30]:

(1) $$E_{r}=A+Be^{-j\varphi_{1}}+Ce^{-j(\varphi_{1}+\varphi_{2})},$$

where the input field (*E*_0_), transmission loss in the cavity (*α*), round trip propagation phase shifts (*ϕ*_1,2_) are given by:

(2) $$\varphi_{1}=\frac{4\pi n_{air}L_{1}}{\lambda },\varphi_{2}=\frac{4\pi n_{smf}L_{2}}{\lambda },$$

(3) $$A=E_{0}R_{1}{}^{1/2},B=E_{0}(1-\alpha )(1-R_{1})R_{2}{}^{1/2},C=E_{0}(1-\alpha )(1-R_{1})(1-R_{2})R_{3}{}^{1/2},$$

(4) $$R_{1}=R_{2}=R_{3}={\left(\frac{n_{smf}-n_{air}}{n_{smf}+n_{air}}\right)}^{2},$$

Using Equations (1)–(4), the total reflection spectrum can be simplified to:

(5) $$I_{r}={\left|A\right|}^{2}+{\left|B\right|}^{2}+{\left|C\right|}^{2}+2AB\cos(\varphi_{1})+2BC\cos(\varphi_{2})+2AC\cos(\varphi_{1}+\varphi_{2})$$

From the above equations: a change in refractive index *n* between *n_smf_* and *n_air_* would change the phase *ϕ* and adding an extra path length along the air for the light to travel would change the spectrum. The reflection spectrum of an FPI is wavelength dependent intensity modulation, generally caused by the optical phase difference between the two beams. The optical path difference caused by the microsphere is given by [31]:

(6) $$OPD_{ms}=n\pi d,$$

where *n* is the microsphere refractive index and *d* is the diameter of the microsphere.

The initial FPI structure (without the microsphere) has been shown previously to be capable of enhancing high-order mode interference, which increases multi-mode interference and improves the intensity change with wavelength shifts (i.e., spectrum slope). The slope of the reflection spectrum given by *S = dR/dλ*, where *S* is the slope and *R* is the reflectivity; this details that a greater slope indicates a stronger multimode interference, which leads to better sensitivity. This corresponds to the spectrum’s quadrature points. The off-core FPI has the capability to add extra modes via the air and silica cladding to enhance the quality factor with an additional one or two round trips, and in turn leads to an improved ultrasound response. The light propagating from the fiber core will also be coupled to the microsphere. The barium in the glass microsphere composition allows for an increase in refractive index to 1.94 (previously mentioned). The higher refractive index allows for the confined light within the microsphere to slow down through this medium allowing for more bends total internal reflection, all of this will lead to more efficient refraction.

## 3. Results and Discussion

**Figure 3:**
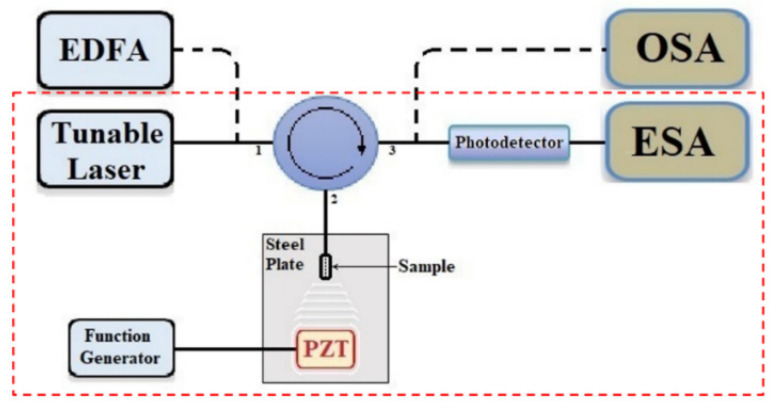
Experimental setup. The ultrasound generation and detection components are indicated within the red dashed box.

**Figure 4:**
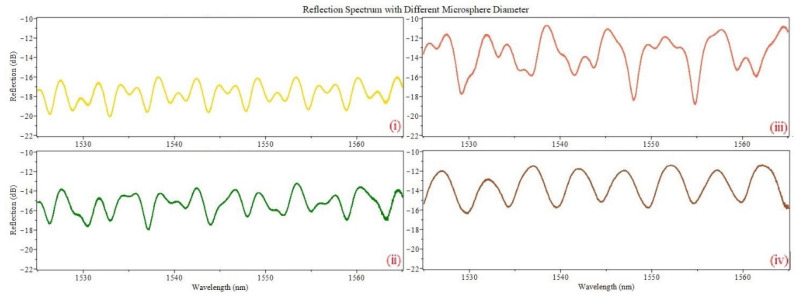
Reflection Spectrum of the same off-core configuration with different microsphere diameters (**i**) 5 µm (**ii**) 10 µm (**iii**) 16 µm (**iv**) 50 µm.

An experimental setup can be seen in Figure 3. The reflection spectrum is obtained through an optical circulator using an erbium-doped fiber amplifier source (INO, Quebec, QC, Canada, FAF-50) and optical spectrum analyzer (Yokogawa, Tokyo, Japan, AQ6375). The reflection spectrum of the microsphere coupled off-core sensor is analyzed under different microsphere diameters, illustrated in Figure 4, which have an offset distance of ~11.4 µm. Different microsphere diameters yield different reflection spectrum for a specific offset. The microsphere diameter and core offset distance play a direct contribution to the reflection contrast. This enhanced contrast and reflectivity determine the dynamic range and signal strength of this device. When the input light from the main fiber core travels through the first air–silica boundary into the open cavity it will meet the microsphere; this leads to a corresponding large number of reflections within the microsphere, which allows for high-order modes and a large contrast increase. This is heavily dependent on the alignment and core offset. When the microsphere diameter is too large compared to the core offset it leads to large propagation loss and a diminished contrast resulting in weak multi-mode interference. The offset distance *h* should be equal to the radius of the microsphere. This alignment is crucial because the light path from the incoming core will be able to directly interact with the apex point of the microsphere (facing the first silica/air interface) allowing for an increase in reflection and coupling. Since the offset distance is ~11.4 µm, the 5 µm, 10 µm, 16 µm, and 50 µm diameter microspheres are not ideal. The ideal microsphere for this specific offset distance would be with a 22.8 µm diameter.

**Figure 5:**
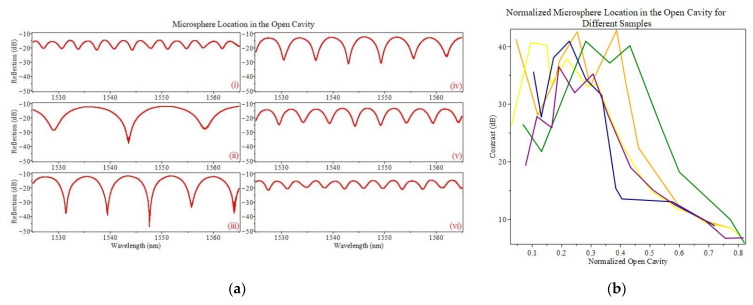

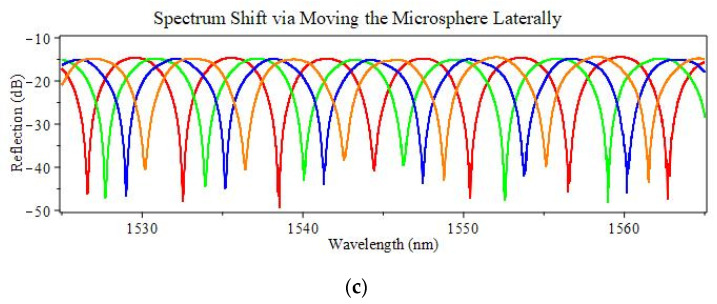
(**a**) Microsphere location with respect to the first silica–air interface (start of the open cavity) (i) No microsphere (ii) 50 µm (iii) 100 µm (iv) 150 µm (v) 200 µm (vi) 250 µm (**b**) Normalized microsphere location for samples with different open cavity lengths with an offset of ~11–12 µm and an optimized microsphere diameter of ~22 µm. (**c**) a full FSR spectrum shift via moving the microsphere laterally at a fixed longitudinal position.

Figure 5a depicts the change in the reflection spectrum as the microsphere distance from the first silica–air interface increases (along the center) for a constant open cavity segment length *L*_1_. As the distance between the beginning of the cavity and microsphere increases, the free spectral range of the reflection decreases. It is determined that the microsphere location with the highest contrast is located between the first ⅓ to ½ distance of the off-core open-air segment. Figure 5b shows the test repeated with different samples (with relatively the same h), changing *L*_1_ to ensure its consistency where the distance is normalized with respect to the open-air cavity length. Once the microsphere is positioned longitudinally (along the fiber direction), it can be moved by a few micrometers (much smaller than the diameter of the microsphere, which is 22 µm, to ensure low loss from leaky modes) laterally (perpendicular to the fiber direction) to display a full FSR shift, which can be seen in Figure 5c. This shift will generally maintain the same shape and FSR of the spectrum due to the FP cavity between the off-core fiber and microsphere, allowing further tunability.

**Figure 6:**
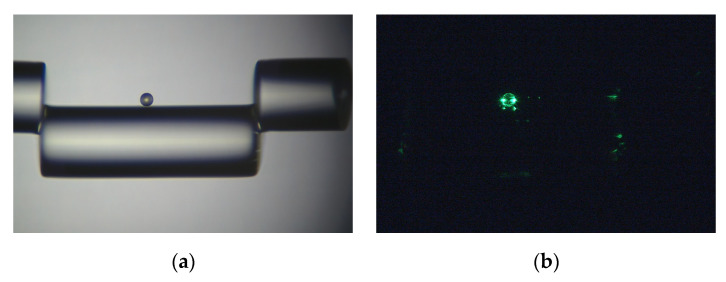
(**a**) Microscope image of the fabricated sample described in Table 1. (**b**) Microscope image of green light passing through the sensor to help with alignment.

**Figure 7:**
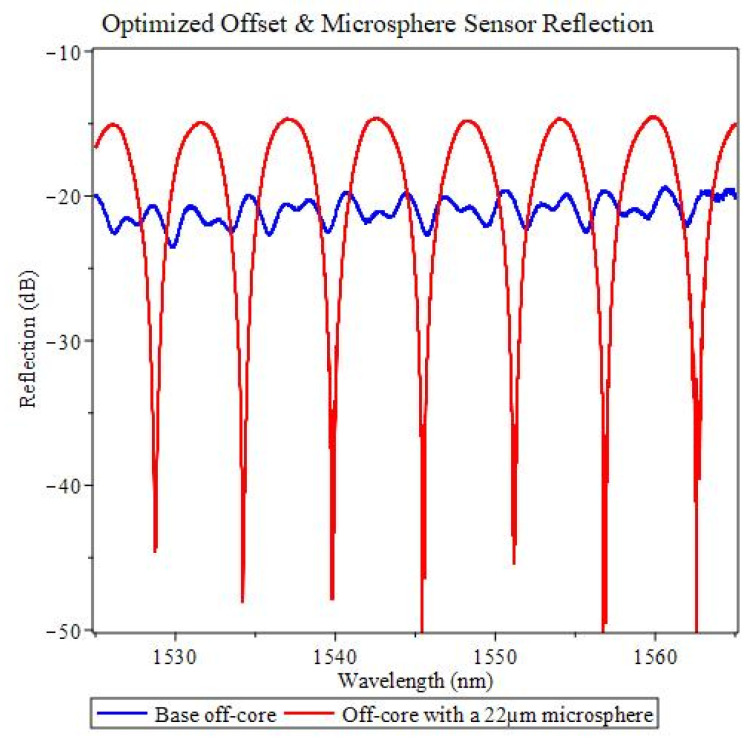
Optimized offset and microsphere diameter reflection spectrum.

Analyzing the data given in both Figure 4 and Figure 5, a sample is fabricated seen in Figure 6a, ensuring the offset distance and the microsphere radius is comparable, and is located between ⅓ and ½ of the total open cavity distance. A green light is sent through the fiber to confirm the offset and microsphere are aligned (Figure 6b). The specific sensor parameters for the device under the test can be seen in Table 1. As a result of the multibeam interference, enhanced finesse via an increased number of FPIs and strong dips in the reflection spectrum allow for a broad tuning range, typically associated with higher sensitivity. The reflection spectrum and its initial spectrum before adding the microsphere are shown in Figure 7. A maximum contrast of ~44 dB can be achieved, which is a drastic increase in contrast when comparing it to its initial off-core case with no microsphere of ~4.2 dB, an increase of over 10 times. If we suppose a single pass FPI (without the microsphere), the finesse is 4, corresponding to the blue spectra in Figure 7, with 6 passages, the new finesse is 4^6^ which is equivalent to 4096, 30 dB. Although, with the multiple surface reflections due to the high reflective index within the microsphere, the new finesse is 4^8^, 45 dB, which has an enhancement factor close to the red spectra.

**Figure 8:**
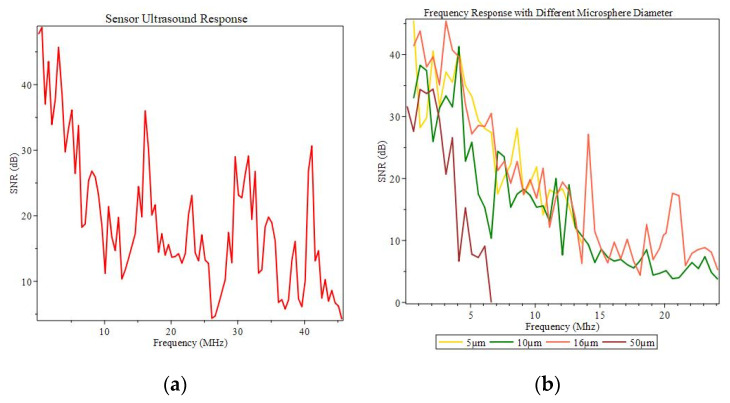
(**a**) Ultrasound response (**b**) Ultrasound response with mismatched offsets and microsphere size.

To test the device for its ultrasound sensing response, a piezoelectric transducer (PZT) fixed to a thin steel plate (0.25 mm thickness) is used as the ultrasound source driven by a function generator (Agilent, Santa Clara, CA, USA, 33250A) (seen previously in Figure 1 boxed in red). A tunable laser source (Agilent 81940A) is set to the specific wavelength 1545.7 nm, which corresponds to a quadrature point in the reflection spectrum for its steep slope and large contrast. It should be noted, although a large contrast is desirable for improved sensitivity, a steep slope plays a more crucial role. The locked probe wavelength can respond to the periodic modulation from the propagating ultrasound waves, which can be analyzed through an electronic spectrum analyzer (ESA) (Rohde & Shwarz, Columbia, MD, USA, F&W Signal Spectrum Analyzer) via a photodetector (Thorlabs, Newton, NJ, USA, PDB45QC-AC). The device under test is encapsulated inside of an acrylic case for added protection from external disturbances, and the ESA is placed in a separate room separated by a concrete wall to eliminate any sort of antenna effect from the ultrasound generation. Figure 8a shows the sample’s frequency response from 0.1–45.6 MHz. The importance of selecting a proper quadrature point is crucial to the device’s ultrasound detection sensitivity. Figure 8b depicts the ultrasound response of an unideal offset-microsphere pairing tested to highlight its importance. Different quadrature points correspond to different maximum frequency responses. This is due to the different resonant modes in the microcavity and its multimode interference. This device can be compared to Fan et al. [26,28], where the microsphere is glued to the off-core fiber and without a microsphere at all, which limited the tuning range; numerous samples were required due to the fixed nature of the off-core FPI cavity to optimize the spectral contrast, which limited the specific contrast, reflectivity, and linewidth. With the flexible lay sphere in our approach, one sample is adequate to optimize the spectrum for maximum contrast in the reflection spectra. If the microsphere radius equals the offset distance, the spectrum can be aligned at quadrature points for ultrasound sensing measurements with the highest sensitivity.

## 4. Conclusions

This paper gave an overview and background of a proposed extrinsic FPI sensor enhanced by multiple pass FPI due to a BaTiO_3_ glass microsphere coupled to the open cavity of a two segment SMF off-core device. The light coupled within the microsphere allows for scattering and internal reflection, which enhances the finesse. Offset alignment of the main incoming SMF and the center of the microsphere is imperative for maximum slope and contrast in the reflection spectrum, which allows for ideal quadrature points to be determined that are best suited for ultrasound detection. This simple and inexpensive sensor offers more applications beyond ultrasound sensing, including refractive index sensing, temperature monitoring, and biological sensing.

## Figures and Tables

**Table 1:** Sensor device specifications.

Specification	
Offset (h)	11.4 µm
1st Segment Length (*L*_1_)	381 µm
End Segment Length (*L*_2_)	155 µm
BaTiO_3_ Diameter	22 µm

## Data Availability

The data presented in this study are available upon request from the corresponding author.
